# The Challenge for Gene Therapy: Innate Immune Response to Adenoviruses

**DOI:** 10.18632/oncotarget.231

**Published:** 2011-03-05

**Authors:** Bart Thaci, Ilya V. Ulasov, Derek A. Wainwright, Maciej S. Lesniak

**Affiliations:** The Brain Tumor Center, The University of Chicago Pritzker School of Medicine, 5841 South Maryland Ave, M/C 3026, Chicago, IL 60637

**Keywords:** Adenovirus, interferon, interleukin-1, innate immune response

## Abstract

Adenoviruses are the most commonly used vectors for gene therapy. Despite the promising safety profile demonstrated in clinical trials, the efficacy of using adenoviruses for gene therapy is poor. A major hurdle to adenoviral-mediated gene therapy is the innate immune system. Cell-mediated recognition of viruses via capsid components or nucleic acids has received significant attention, principally thought to be regulated by the toll-like receptors (TLRs). Antiviral innate immune responses are initiated by the infected cell, which activates the interferon (IFN) response to block viral replication, while simultaneously releasing chemokines to attract neutrophils, mononuclear- and natural killer-cells. While the IFN and cellular recruitment pathways are activated and regulated independently of each other, both are required to overcome immune escape mechanisms by adenoviruses. Recent work has shown that the generation of adenoviral vectors lacking specific transcriptionally-active regions decreases immune system activation and increases the chance for immune escape. In this review, we elucidate how adenoviral vector modifications alter the IFN and innate inflammatory pathway response and propose future targets with clinically-translational relevance.

## INTRODUCTION

Adenoviruses are a continuously expanding class of at least 51 immunologically distinct serotypes [1] classified into 6 species (A-F) based on their hemagglutination properties, oncogenic potential, genotyping and phylogenetic analyses [2]. A small number of viral particles are sufficient to induce symptoms that will be determined by the adenovirus type and the inflammatory mediators [3]. In immunocompetent hosts, adenoviruses cause mild ocular, respiratory and gastrointestinal tract diseases. Thus, the safety profile for adenoviral administration into animals has been one of the reasons for their widespread use as gene therapy vectors. Although local injections have proven to be safe in clinical trials, intravascular delivery has been associated with one reported fatality [4]. An acute inflammatory response to the adenoviral vector was later determined to be the cause of the fatality. Therefore, investigating the complex interplay between adenoviruses and the immune system is of paramount importance for future safe and effective gene therapy.

## ADENOVIRUS BIOLOGY

**Figure 1:**
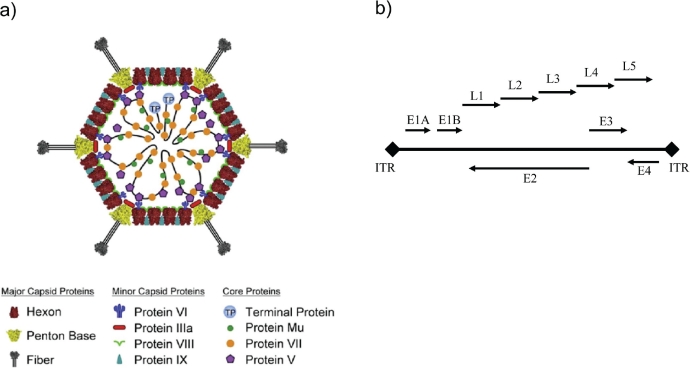
Adenovirus structure and transcription regions *A*) Adenovirus capsid is composed of three major and four minor proteins. The three major proteins plus protein IX form the outer surface. Reproduced with permission [5] *B*) The double stranded DNA adenoviral linear genome is covalently linked at its ends to terminal proteins via inverted terminal repeats (ITRs). The first viral transcription unit to be expressed is E1A that activates the other early adenovirus transcripts (E1B, E2, E3 and E4). Late gene transcription (L1-L5) is activated after the onset of DNA replication and is under the control of major late promoter (MLP). Arrows indicate the direction of transcription.

The adenovirus is a dsDNA non-enveloped virus of 70-90nm in size (Figure 1A). Its core, a 36kB double-stranded linear DNA is packed within the icosahedral capsid [5]. The viral genome encodes several early (E1A, E1B, E2, E3 and E4) and late (L1-5) transcriptional units (Figure 1B) that give rise to multiple mRNAs and proteins via differential processing [6]. The capsid proteins are the primary antigens that define the various serotypes. Hexon- and penton-subunits form the icosahedral ‘shell’, while fiber protrusions help the virus to anchor to the cell surface.

### Adenoviral transduction

Initial attachment of virion particles is mediated by the interaction of the fiber knob domain with the Coxsackie-Adenovirus Receptor (CAR) for all groups beside B, which uses CD46 [7, 8]. Internalization, via clathrin-dependent endocytosis, is mediated by a secondary interaction of the RGD motif on the penton base with αv integrins on the cell surface [9]. While endosomal escape is known to be dependent on the acidity of the microenvironment, the molecular steps that govern viral escape remain to be elucidated. Once in the cytoplasm, virion trafficking is guided by dynein along microtubules, which docks to the nuclear pore complex where viral DNA enters the nucleus [10, 11]. All of the steps including viral attachment and intracellular escape, as well as DNA transcription, take only 10 minutes [12].

## THE INTERLEUKIN-1/INFLAMMATORY PATHWAY

The complexity of the innate immune response is matched only by the diversity of experimental designs that are used to investigate it. Different routes of delivery, conditions, time points and targets make this complicated system even more difficult to understand, which includes high levels of redundancy and cell-type specificity [12, 13]. Thus, understanding how all of these seemingly different responses and measurable variables are related is required.

In this review, we offer a more structured approach to understanding the innate immune response as a result of adenoviral entry by dissecting the major pathways involved. Of the many independent receptors in innate immunity, two stand out: the interferon α receptor (IFN-AR) and interleukin-1 receptor (IL-1R) The downstream effectors of IFN-AR and IL-1R diverge to block viral replication using different signal transduction pathways. While the IFN response acts in an autocrine/paracrine fashion to contain and destroy the virus from within the cell, the interleukin pathway recruits a pro-inflammatory infiltrate to eliminate the pathogen.

The quick nature of the inflammatory responses suggests that an early recognition of the virus activates an immature form of IL-1 which resides in the cytosol. But the maximum inflammatory response relies on a fully-functioning IL-1R [12, 14] and a number of protein kinases induced upon infection (Figure 1). These protein kinases, JNK, ERK1/2 and p38 MAPK, are also downstream effectors of non-specific stress response pathways [15].

**Figure 2:**
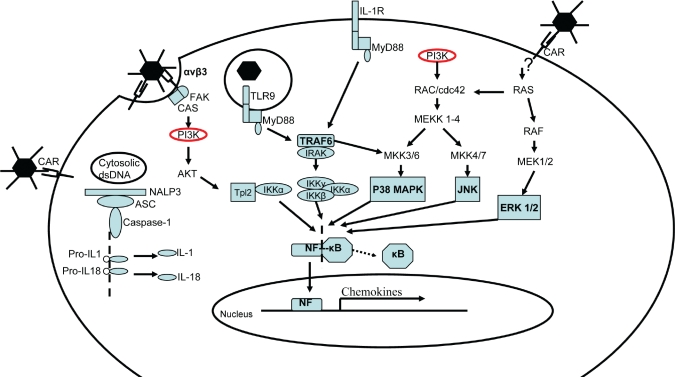
Inflammatory response to adenovirus infection CAR or integrin binding to adenovirus triggers PI3K and MAPK kinases. NLRs activate IL-1/18 which act via IL-1R to induce IKK kinases, which are also induced by recognition of viral DNA in endosomes. After kinases release NF from NF-κB, it transfers to the nucleus to induce chemokine transcription.

The earliest sensor to be activated upon viral infection appears to be triggered by the adenoviral fiber binding with the CAR [16]. While the downstream signaling of CAR has yet to be elucidated, recent work has shown that CAR promotes the clustering of junctional adhesion molecule-like protein (JAML) and activation of phosphoinositide 3-kinase (P13K) [17]. Irrespective of the possible effectors involved [18], adenovirus binding to CAR induces downstream signaling of ERK1/2, JNK and MAPK, followed by NF-κB activation (Figure 2) and the up-regulation of chemokines.

Current dogma supports the hypothesis that the earliest event inducing innate immunity is the interaction of the RGD motif with αv integrins, especially B3 [12]. Furthermore, the downstream signaling of αv integrins induces NF-κB activation and subsequently increases IL-1 expression, which has been reviewed elsewhere [19]. Importantly, the intensity of this response depends on endosomal viral escape and the presence of foreign DNA, since empty capsids do not induce transcription of chemokines [13].

Once internalized within host cells, adenoviral dsDNA can be sensed within the endosome via several TLRs [20] such as TLR9; in the cytosol by DNA-dependent activator of IFN-regulatory factors (DAI) and/or nucleotide oligomerization domain (NOD)-like receptors (NLRs). Thus, the context of the immune response will depend on which of the pathways become activated. Downstream effectors of activated TLR9 diverge to activate both IFN- and inflammatory-responses. Accordingly, DAI relies on the IκB kinases, TBK1/IKKi, to induce the IFN response [21]. In contrast, NLRs bind with apoptosis speck protein (ASC) and caspases to form the ‘inflammasome’ [22], which cleaves the immature forms of pro-IL-1 and pro-IL-18 into their mature and active forms [23]. Subsequently, IL-1R activation leads to activation of NF-κB followed by the release of various chemokines (Table 1) [12, 16, 18, 24-29]. The broad effects of chemokines induced during adenoviral delivery have to be considered when designing gene therapy vectors. The resulting immune derangements, ranging from immunosuppression to autoimmunity, play a significant role in pathogenesis. The ability to decrease the level of inflammation [30] or enhance and modulate the immune response [31] with adenoviral vectors will increase the potential for immunotherapeutic success.

**Table 1:** Triggered innate inflammatory pathways

Chemokine	Triggered pathway	Reference
IL-1	The ‘endogenous pyrogen’, induces production of all the other cytokines	12
IL-18	Enhances T and NK cell maturation, cytokine production and cytotoxicity	25
IL-8	Polymorphonuclear leukocyte chemotaxis and activation	26
GRO-α,γ	Structurally and functionally similar to IL-8	16
IL-6	Secreted by monocytes and macrophages during adenovirus infection	24
CXCL1	Induces neutrophil chemotaxis and respiratory burst activity	27
MCP-1	Monocyte and T-lymphocyte chemotactic factor	28
RANTES	Chemotactic factor for NK cells	29
TNF-α	Triggers apoptosis or necrosis in infected cells	18

## INTERFERON/PARACRINE RESPONSE

Three classes of IFNs have been identified according to the receptor through which they signal. Type I IFNs comprise 13 IFN-α subtypes, IFN-β, IFN-κ, IFN-ε, IFN-ο, IFN-τ and IFN-δ. All of the type I IFNs signal through IFN-AR. Thus, mice deficient for IFN-AR have an increased susceptibility to viral infections [32]. Type II IFNs are secreted by lymphocytes in response to different pathogens during the adaptive immune response, while type III IFNs are not well characterized [33]

Adenovirus-mediated IFN responses are induced, at least in part, by the recognition of foreign nucleic acid. Unlike the inflammatory responses, IFN has not been shown to be induced during adenoviral interaction with cell-surface receptors. Furthermore, the adenovirus-induced IFN response is cell specific [34]. While myeloid dendritic cells (mDCs) recognize adenoviral DNA in the cytoplasm, plasmacytoid DCs (pDCs) depend on TLR9, located in endosomes.

**Figure 3:**
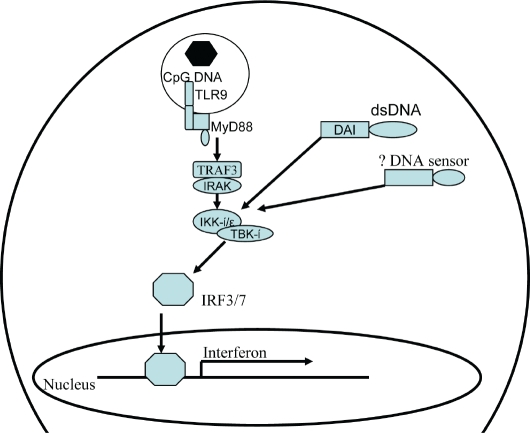
Interferon induction during adenovirus infection In plasmacytoid DC adenoviral DNA activates TLR9 in plasmacytoid DCs, whereas DAI is activated in mDCs. The down-stream signaling converges in TBK1/IKKί activation and culminates in IRF3/7 transfer to the nucleus to induce IFN transcription.

The discovery of the TLRs resulted in a new and focused investigative path for innate immunity [35, 36]. The mammalian TLR family consists of at least 11 members localized on the cell surface or inside endosomes. Important to adenoviral recognition, TLR9 localizes within endosomes and recognizes adenoviral CpG-rich DNA. In response to TLR9-activation, pDCs secrete IFN-α via the MyD88-dependent pathway [37]. The MyD88-dependent pathway is analogous to the IL-1R pathway: after stimulation, MyD88 recruits IL-1 receptor associated receptor kinase 1 (IRAK1) and TNF receptor-associated factor-6 (TRAF6). This complex activates MAP kinases and interferon regulatory factor 7 (IRF7). In pDCs, this pathway leads to the production of type I IFNs [38] (Figure 3), whereas in other types of DCs, such as mDCs, it leads to NF-κB activation and subsequent increased pro-inflammatory cytokine expression.

In mDCs, macrophages or fibroblasts, IFN production is not related to TLR recognition, nor dependent on the MyD88/TRIF pathway [37]. Rather, the presence of a cytosolic receptor, DAI, is activated and subsequently induces an IFN response upon foreign DNA recognition [21]. The downstream effectors of DAI are dependent on TBK1/IKKi and IRF3/7 for type 1 IFN induction. However, the recent generation of DAI-KO mice has contradicted previous data in the field, which demonstrates that DAI expression is not essential for the IFN response. Whether the MyD88/TRIF and DAI pathways are redundant in some cells, or other receptors exist, is currently unknown.

The aforementioned pathways that lead to the induction of IFN expression are not affected by adenovirus. Some viruses such as hepatitis C virus (HCV) or varicella-zoster virus (VZV) have been demonstrated to prolong viral replication in tissues by inhibiting type I IFN transcription [39, 40]. In contrast, type I IFN expression is not affected by adenoviral infection. However, the downstream pathways of IFN expression are blocked. Regardless, once induced, IFN acts in a paracrine/autocrine fashion to block adenoviral replication.

Type I IFNs bind to their cognate receptor which leads to the activation of JAK1/TYK2 kinases and is followed by the recruitment and phosphorylation of STATs. STAT1 and STAT2 associate with IRF9 to form ISGF3, which induces more than 300 IFN-stimulated genes (ISGs) [41]. The intensity of this response is drastically reduced during adenoviral infection, which inhibits the formation of ISGF3. As a result, only a few ISGs, such as Protein Kinase R (PKR) and Myxovirus resistance A protein (MxA) [42] have been shown to be activated upon adenoviral infection. PKR belongs to a family of protein kinases that respond to environmental stresses by phosphorylating E1F2α to regulate protein synthesis. At the N-terminus of PKR, two RNA binding motifs exist that release the negative steric hindrance and allow dimerization of PKR on contact with RNA [43]. In contrast, MxA GTPases are involved in vesicle budding, organogenesis and cytokinesis. By virtue of their location near the endoplasmic reticulum, MxA can block viral replication at early time points by trapping essential viral proteins [44].

A complex interplay between the IFN and inflammatory responses is necessary to clear adenoviral infections. In bone marrow-derived macrophages, IRF3 activation and IFN induction have been shown to be influenced by inhibitors of the inflammatory pathway, such as JNK inhibitors [45]. Interaction of integrins with the adenoviral fiber RGD motif induces JNK to prime IRF3, whose full activation requires phosphorylation by TBK1. In addition, the recruitment of NK cells into the liver after intravenous injection of adenovirus is independent of the IFN response. However, the activation of NK cells requires IFN downstream signaling, since recruited NK cells in IFN-R^−/−^ mice fail to control adenoviral infection [46].

Through evolution, adenoviruses have developed mechanisms to dampen and escape the immune response. These are more effective against ISGs than inflammatory mediators. Moreover, during adenoviral infections, inflammatory mediator levels such as IL-1α increase 20-fold, while IFN-α increases less than 2-fold [3]. However, genetic manipulation of adenovirus is independent of the IFN response. More importantly, generation of adenoviruses lacking specific innate immune system-interfering genes has allowed the mechanistic study and determination of relevance for many genes required for innate immune system-mediated adenoviral clearance [47].

### Immune Response to Adenoviral Vectors

Adenoviruses are the most commonly used gene therapy vectors. Due to the small size, the adenoviral capsid structure does not allow for extra DNA to be packaged properly. Thus, in order to insert a gene of interest into the adenoviral genome, a similar size of viral DNA must be removed. Furthermore, the viral cytopathic effect has to be minimized. The first generation E1-deleted adenoviral vectors were less cytopathic but induced innate immune responses very quickly [28, 48]. To decrease overall immune activation, vectors were deleted of more dispensable transcriptional regions. Three generations of vectors were created. Of those, ‘gutless’ vectors, deleted for the entire adenoviral genome, had the least antigenic and immunostimulatory properties.

Adenoviral vectors deficient of early transcript regions showed a different sensitivity towards IFN and inflammatory responses. Deletion of the E1 region reduced the IFN resistance more than deleting E4. Furthermore, vectors lacking E3 genes were more vulnerable to inducing the inflammatory response. It should be noted that the E2 transcripts have not been shown to block the innate immune response. Rather, E2 gene transcripts boost the immune response [49].

Deletion of E1A has been shown to be very important for inhibiting the immune response. The products of E1A, 289R and 243R, named after their amino acid residue length, not only bind to cell cycle regulatory proteins such as pRB, but also have transcriptional activator/repressor properties. It is clear that E1A can rescue other viruses from the IFN response by inhibiting the formation of the ISGF3 complex [50, 51]. For example, E1A inhibits transcription of JAK1 in epithelial cells [42]. In contrast, the E1B region encodes three major proteins that are 55-kDa, 19-kDa and 17-kDa in size. The components of E1B, complexed with the E4-ORF6, block host mRNA transport and p53 transcriptional activity while facilitating late viral mRNA expression. Furthermore, the E1B-19-kDa component is a BCL2 homologue and acts as a potent inhibitor of apoptosis.

Adenoviral transduction of cells results in the transcription of both host and viral DNA, resulting in a greater amount of total DNA within a cell, which can trigger host cell defenses. However, the adenovirus has selectively evolved to deal with those cell defenses. E4 proteins hijack the machinery associated with the DNA damage pathways [52]. There are six E4 proteins produced through differential splicing of the open reading frame (ORF). The E4ORF3 and E4ORF6 show functional redundancy. Both form complexes with the E1B-55kDa component and regulate late adenoviral gene expression.

Inflammatory responses induced by adenoviral transduction are also counteracted by the E3 region transcripts. Inserting the E3 region in recombinant vectors decreases the maximal innate immune response, which consequently permits long term adenoviral gene expression [53]. The E3-14.7 kDa component inhibits the NF-κB-induced transcription of inflammatory mediator expression [54], while the E3-10.4kDa/14.5kDa components inhibits TNFα and FAS ligand-induced cell death by internalizing receptor internalization and degradation alpha/beta (RIDα/β) receptors [55]. The E3-19kDa glycoprotein inhibits peptide presentation by MHC class I via a specific motif at the carboxy terminus, which retargets MHC class I to the endoplasmic reticulum [56].

The functional relevance of adenoviral protein interference with innate immunity must be carefully understood if an effective therapy can be produced. For gene therapy, which requires the prolonged expression of therapeutic genes by a transduced cell, maintenance of cell viability and the reduction of inflammation is required. The E3-14.7kDa component inhibits the NF-κB-induced transcription of inflammatory mediator expression [54], while the E3-10.4kDa/14.5kDa component inhibits TNFα and FAS ligand-induced cell death by receptor internalization and degradation of alpha/beta (RIDα/β) receptors [55]. In addition, inflammation can be further reduced by immunosuppressive drugs, such as cyclophosphamide, which have been shown to enhance the effectiveness of adenoviral vectors in different species in non-toxic doses [58, 59].

The overall intensity of the systemic immune response will depend on the level of induced inflammatory mediators by the infected cell. Fiber modifications have expanded the spectrum of cells that adenoviruses can infect, which consequently increases the risk of adverse effects, especially when injected systemically. However, intravascular delivery of gene vectors is required for effectively treating metastatic disease or the transduction of large number of cells *in vivo*.

### Intravascular delivery

Irrespective of the fiber modifications, adenoviruses are sequestered by liver cells after intravascular injection [60]. When the liver is not the direct target, intravascular delivery of naked virus has been associated with adverse side-effects and limited efficacy. However, the discovery of coagulation factor binding properties of virions and differences between adenoviral groups has elucidated the mechanism to avoid for liver detargeting [61, 62]. Of the different strategies used to limit the toxicity of adenoviral vectors during systemic administration, only a handful have been beneficial including surface modifications, genetic deletion and use of cell carriers.

Surface modifications have been of great interest not only for intravascular delivery but also for improving cell transduction since wild-type adenovirus fibers cannot transduce target cells lacking CAR. However, fiber modifications alone [24] have not proven to limit the binding with coagulation factors, such as FX or FIX. Structural and mutagenesis studies have shown Ad5 hexon hypervariable regions (HVR) 5 and 7 to be critical for this interaction [63]. To prevent this interaction, genetic modifications, hexon serotype substitution and PEG-masking are useful approaches with proven success, while maintaining efficacy. By genetically inserting a large amino acid biotin acceptor peptide (BAP) into HVR5, affinity to FX factor was reduced 10,000-fold. This allowed a 10-fold increase in the maximum tolerated dose [64]. Substitution of Ad5 hexon with serotype 3 had very similar effects [65]. Thus, hexon modifications promote immune escape, which is critical to successful therapy since most patients already possess circulating antibodies to Ad5 hexon [66].

Polyethylene glycol (PEG) is a linear synthetic polymer that can be synthesized to varying lengths, from 200-40,000D. Its properties include low toxicity, low immunogenicity and hydrophilicity, making it a very attractive compound for modifications of various peptides or proteins. This has also proven true for adenovirus PEGylation [67]. The PEG 20kDa length has proven to be most effective [68, 69]. Decreased activation of the immune response and reduced IL-6 expression was inversely correlated with the PEGylation rate when compared to native Ad5 [70, 71].

Hepatic detargeting can also be achieved using cells as vehicles for adenovirus delivery, instead of ‘naked’ adenovirus [72]. Our lab has shown that the inflammatory response is reduced by using mesenchymal stem cells loaded with adenoviruses [73]. These immunosuppressive properties can therefore be harnessed to achieve long-term transgene expression. Also, stem cells loaded with adenoviral vectors have shown to be attracted to growth factors or chemokines and can target specific niches even when injected systemically [74].

Our rationale for investigating and exploring the adenovirus for gene therapy is to further increase the efficacy for potential clinical use in glioblastoma patients. One such way to do this is by using a surface modification combining PEGylation with liposome encapsulation of the adenovirus [75]. This has been performed with success using systemically-delivered PEGylated adenovirus with tumor cell-specific promoters to control metastasis [69]. However, as we investigate new mutated or aberrantly expressed tumor promoters, more effective adenoviral vectors will be constructed.

## CONCLUSIONS

The innate immune response to adenoviral vectors will always interfere with gene therapy in some way. During each step of viral delivery there is a barrier to overcome. Many different sensors exist for adenoviral replication starting from the initial attachment to the cell surface via the CAR and integrins, later in endosomes via TLRs, in the cytoplasm and even in the nucleus. Modifications have made adenoviral vectors even more sensitive to innate immune responses. Understanding these different pathways and how to modulate them will bring us closer to clinical use. Cell specificity appears to be a major limitation in predicting human innate immune responses. The unpredictable adverse effects encountered during intravascular therapy should not make us bitter about the potentials of adenoviral vectors. Experience with ‘gutless’ vectors has proven that less is more, even in medicine.
